# Supplementary material for: The Deuterium Oxide Dilution Method to Quantify Human Milk Intake Volume of Infants: A Systematic Review—A Contribution from the ConcePTION Project
Source: Nutrients. 2024 Dec 5;16(23):4205. doi: 10.3390/nu16234205 (PMC11644218; doi:10.3390/nu16234205)
Supplement: Supplementary file 1 [file nutrients-16-04205-s001.zip › nutrients-3347538-supplementary materials.pdf]

Supplementary materials

Table S1: Search string of PubMed, Embase, Web of science, Cochrane library, Scopus and CINAHL

Concept 1	
**PubMed**	"Deuterium Oxide"[Mesh] OR "Deuterium"[Mesh] OR "deuteriumoxide"[tiab] OR "heavy water"[tiab] OR "heavywater"[tiab] OR "deuterated water"[tiab] OR "deuteratedwater"[tiab] OR "deuterated oxide"[tiab:~0] OR "deuteratedoxide"[tiab] OR "D2O"[tiab] OR "2H2O" [tiab] OR “D2 oxide”[tiab:~0] OR “2H oxide”[tiab] OR "deuterium"[tiab] OR "hydrogen 2"[tiab] OR "hydrogen2"[tiab] OR "deuteron*"[tiab] OR "heavy hydrogen"[tiab] OR "heavyhydrogen"[tiab] OR "hydrogen oxide h 2"[tiab:~0] OR "hydrogen oxide h2"[tiab:~0] OR "hydrogenoxide h2"[tiab:~0] OR "water h 2"[tiab] OR "water h2"[tiab] OR "hydrogen h 2"[tiab] OR "hydrogen h2"[tiab]
**Embase**	'deuterium oxide'/exp OR 'deuterium'/exp OR 'deuteriumoxide':ti,ab,kw OR 'heavy water':ti,ab,kw OR 'heavywater':ti,ab,kw OR 'deuterated water':ti,ab,kw OR 'deuteratedwater':ti,ab,kw OR 'deuterated oxide':ti,ab,kw OR 'deuteratedoxide':ti,ab,kw OR 'D2O':ti,ab,kw OR '2H2O':ti,ab,kw OR 'D2 oxide':ti,ab,kw OR '2H oxide':ti,ab,kw OR 'deuterium':ti,ab,kw OR 'hydrogen 2':ti,ab,kw OR 'hydrogen2':ti,ab,kw OR 'deuteron*':ti,ab,kw OR 'heavy hydrogen':ti,ab,kw OR 'heavyhydrogen':ti,ab,kw OR 'hydrogen oxide h 2':ti,ab,kw OR 'hydrogen oxide h2':ti,ab,kw OR 'hydrogenoxide h2':ti,ab,kw OR 'water h 2':ti,ab,kw OR 'water h2':ti,ab,kw OR 'hydrogen h 2':ti,ab,kw OR 'hydrogen h2':ti,ab,kw NOT ‘conference abstract’:it
**Web of science**	TS=("deuteriumoxide" OR "heavy water" OR "heavywater" OR "deuterated water" OR "deuteratedwater" OR "deuterated oxide" OR "deuteratedoxide" OR "D2O" OR "2H2O" OR "D2 oxide" OR "2H oxide" OR "deuterium" OR "hydrogen 2" OR "hydrogen2" OR "deuteron*" OR "heavy hydrogen" OR "heavyhydrogen" OR "hydrogen oxide h 2" OR "hydrogen oxide h2" OR "hydrogenoxide h2" OR "water h 2" OR "water h2" OR "hydrogen h 2" OR "hydrogen h2") NOT DT=("meeting abstract”)
**Cochrane library**	#1: [mh "Deuterium Oxide"] OR [mh "Deuterium"]<br>#2: ("deuteriumoxide" OR "heavy water" OR "heavywater" OR "deuterated water" OR "deuteratedwater" OR "deuterated oxide" OR "deuteratedoxide" OR "D2O" OR "2H2O" OR "D2 oxide" OR "2H oxide" OR "deuterium" OR "hydrogen 2" OR "hydrogen2" OR deuteron* OR "heavy hydrogen" OR "heavyhydrogen" OR "hydrogen oxide h 2" OR "hydrogen oxide h2" OR "hydrogenoxide h2" OR "water h 2" OR "water h2" OR "hydrogen h 2" OR "hydrogen h2"):ti,ab,kw<br>#3: #1 OR #2
**Scopus**	TITLE-ABS("deuteriumoxide" OR "heavy water" OR "heavywater" OR "deuterated water" OR "deuteratedwater" OR "deuterated oxide" OR "deuteratedoxide" OR "D2O" OR "2H2O" OR "D2 oxide" OR "2H oxide" OR "deuterium" OR "hydrogen 2" OR "hydrogen2" OR "deuteron*" OR "heavy hydrogen" OR "heavyhydrogen" OR "hydrogen oxide h 2" OR "hydrogen oxide h2" OR "hydrogenoxide h2" OR "water h 2" OR "water h2" OR "hydrogen h 2" OR "hydrogen h2") OR AUTHKEY("deuteriumoxide" OR "heavy water" OR "heavywater" OR "deuterated water" OR "deuteratedwater" OR "deuterated oxide" OR "deuteratedoxide" OR "D2O" OR "2H2O" OR "D2 oxide" OR "2H oxide" OR "deuterium" OR "hydrogen 2" OR "hydrogen2" OR "deuteron*" OR "heavy hydrogen" OR "heavyhydrogen" OR "hydrogen oxide h 2" OR "hydrogen oxide h2" OR "hydrogenoxide h2" OR "water h 2" OR "water h2" OR "hydrogen h 2" OR "hydrogen h2")
**CINAHL**	TI ("deuteriumoxide" OR "heavy water" OR "heavywater" OR "deuterated water" OR "deuteratedwater" OR "deuterated oxide" OR "deuteratedoxide" OR "D2O" OR "2H2O" OR "D2 oxide" OR "2H oxide" OR "deuterium" OR "hydrogen 2" OR "hydrogen2" OR "deuteron*" OR "heavy hydrogen" OR "heavyhydrogen" OR "hydrogen oxide h 2" OR "hydrogen oxide h2" OR "hydrogenoxide h2" OR "water h 2" OR "water h2" OR "hydrogen h 2" OR "hydrogen h2") OR AB ("deuteriumoxide" OR "heavy water" OR "heavywater" OR "deuterated water" OR "deuteratedwater" OR "deuterated oxide" OR "deuteratedoxide" OR "D2O" OR "2H2O" OR "D2 oxide" OR "2H oxide" OR "deuterium" OR "hydrogen 2" OR "hydrogen2" OR "deuteron*" OR "heavy hydrogen" OR "heavyhydrogen" OR "hydrogen oxide h 2" OR "hydrogen oxide h2" OR "hydrogenoxide h2" OR "water h 2" OR "water h2" OR "hydrogen h 2" OR "hydrogen h2")

Concept 2	
**PubMed**	"Milk, Human"[Mesh] OR "Breast Feeding"[Mesh] OR "Lactation"[Mesh] OR "human milk"[tiab] OR "humanmilk"[tiab] OR "woman milk"[tiab] OR "womanmilk"[tiab] OR "breast milk"[tiab] OR "breastmilk"[tiab] OR "mother milk"[tiab] OR "mothermilk"[tiab] OR "maternal milk"[tiab] OR "maternalmilk"[tiab] OR "breast fe*"[tiab] OR "breastfe*"[tiab] OR "lactati*"[tiab] OR "milk secreti*"[tiab] OR "milksecreti*"[tiab] OR "wet nursing"[tiab] OR "wetnursing"[tiab] OR "breast pump*"[tiab] OR "breastpump*"[tiab] OR "breast secreti*"[tiab] OR "breastsecreti*"[tiab] OR "lactic secreti*"[tiab] OR "lacticsecreti*"[tiab] OR "milk releas*"[tiab] OR "milkreleas*"[tiab] OR "milk excreti*"[tiab] OR "milkexcreti*"[tiab]
**Embase**	'breast milk'/exp OR 'breast feeding'/exp OR ‘breastfeeding’/exp OR 'lactation'/exp OR ‘human milk’:ti,ab,kw OR 'humanmilk':ti,ab,kw OR 'woman milk':ti,ab,kw OR 'womanmilk':ti,ab,kw OR 'breast milk':ti,ab,kw OR 'breastmilk':ti,ab,kw OR 'mother milk':ti,ab,kw OR 'mothermilk':ti,ab,kw OR 'maternal milk':ti,ab,kw OR 'maternalmilk':ti,ab,kw OR 'breast fe*':ti,ab,kw OR 'breastfe*':ti,ab,kw OR 'lactati*':ti,ab,kw OR 'milk secreti*’:ti,ab,kw OR 'milksecreti*’:ti,ab,kw OR 'wet nursing':ti,ab,kw OR 'wetnursing':ti,ab,kw OR 'breast pump*':ti,ab,kw OR 'breastpump*':ti,ab,kw OR 'breast secreti*':ti,ab,kw OR 'breastsecreti*':ti,ab,kw OR 'lactic secreti*':ti,ab,kw OR 'lacticsecreti*':ti,ab,kw OR 'milk releas*':ti,ab,kw OR 'milkreleas*':ti,ab,kw OR 'milk excreti*':ti,ab,kw OR 'milkexcreti*':ti,ab,kw NOT ‘conference abstract’:it
**Web of science**	TS= ("human milk" OR "humanmilk" OR “woman milk" OR "womanmilk" OR "breast milk" OR "breastmilk" OR "mother milk" OR "mothermilk" OR "maternal milk" OR "maternalmilk" OR "breast fe*" OR "breastfe*" OR "lactati*" OR "milk secreti*" OR "milksecreti*" OR "wet nursing" OR "wetnursing" OR "breast pump*" OR "breastpump*" OR "breast secreti*" OR "breastsecreti*" OR "lactic secreti*" OR "lacticsecreti*" OR "milk releas*" OR "milkreleas*" OR "milk excreti*" OR "milkexcreti*") NOT DT=("meeting abstract”)
**Cochrane library**	#3: [mh "Milk, Human"] OR [mh "Breast Feeding"] OR [mh "Lactation"]<br>#4: ("human milk" OR "humanmilk" OR "woman milk" OR "womanmilk" OR "breast milk" OR "breastmilk" OR "mother milk" OR "mothermilk" OR "maternal milk" OR "maternalmilk" OR (breast NEXT fe*) OR breastfe* OR lactati* OR (milk NEXT secreti*) OR milksecreti* OR "wet nursing" OR "wetnursing" OR (breast NEXT pump*) OR breastpump* OR (breast NEXT secreti*) OR breastsecreti* OR (lactic NEXT secreti*) OR lacticsecreti* OR (milk NEXT releas*) OR milkreleas* OR (milk NEXT excreti*) OR milkexcreti*):ti,ab,kw<br>#5: #3 OR #4
**Scopus**	TITLE-ABS("human milk" OR "humanmilk" OR “woman milk" OR "womanmilk" OR "breast milk" OR "breastmilk" OR "mother milk" OR "mothermilk" OR "maternal milk" OR "maternalmilk" OR "breast fe*" OR "breastfe*" OR "lactati*" OR "milk secreti*" OR "milksecreti*" OR "wet nursing" OR "wetnursing" OR "breast pump*" OR "breastpump*" OR "breast secreti*" OR "breastsecreti*" OR "lactic secreti*" OR "lacticsecreti*" OR "milk releas*" OR "milkreleas*" OR "milk excreti*" OR "milkexcreti*") OR AUTHKEY("human milk" OR "humanmilk" OR “woman milk" OR "womanmilk" OR "breast milk" OR "breastmilk" OR "mother milk" OR "mothermilk" OR "maternal milk" OR "maternalmilk" OR "breast fe*" OR "breastfe*" OR "lactati*" OR "milk secreti*" OR "milksecreti*" OR "wet nursing" OR "wetnursing" OR "breast pump*" OR "breastpump*" OR "breast secreti*" OR "breastsecreti*" OR "lactic secreti*" OR "lacticsecreti*" OR "milk releas*" OR "milkreleas*" OR "milk excreti*" OR "milkexcreti*")
**CINAHL**	(MH "Milk, Human+") OR (MH "Breast Feeding+") OR (MH "Lactation") OR TI ("human milk" OR "humanmilk" OR “woman milk" OR "womanmilk" OR "breast milk" OR "breastmilk" OR "mother milk" OR "mothermilk" OR "maternal milk" OR "maternalmilk" OR "breast fe*" OR "breastfe*" OR "lactati*" OR "milk secreti*" OR "milksecreti*" OR "wet nursing" OR "wetnursing" OR "breast pump*" OR "breastpump*" OR "breast secreti*" OR "breastsecreti*" OR "lactic secreti*" OR "lacticsecreti*" OR "milk releas*" OR "milkreleas*" OR "milk excreti*" OR "milkexcreti*") OR AB ("human milk" OR "humanmilk" OR “woman milk" OR "womanmilk" OR "breast milk" OR "breastmilk" OR "mother milk" OR "mothermilk" OR "maternal milk" OR "maternalmilk" OR "breast fe*" OR "breastfe*" OR "lactati*" OR "milk secreti*" OR "milksecreti*" OR "wet nursing" OR "wetnursing" OR "breast pump*" OR "breastpump*" OR "breast secreti*" OR "breastsecreti*" OR "lactic secreti*" OR "lacticsecreti*" OR "milk releas*" OR "milkreleas*" OR "milk excreti*" OR "milkexcreti*")
Concept 1 AND Concept 2	

Table S2: The Newcastle-Ottowa scale (NOS) used for quality assessment of cohort studies [1]

NOS scale		Selection				Comparability		Outcome			
Studies first author	Year	“Representativeness of the exposed cohort”	“Selection of the non exposed cohort”	“Ascertainment of exposure”	“Demonstration that outcome of interest was not present at start of study”	“Comparability of cohorts on the basis of the design or analysis”		“Assessment of outcome”	“Was follow-up long enough for outcomes to occur”	“Adequacy of follow up of cohorts”	Score
Caire-Juvera et al. [2]	2012	*	*	*	*	*	0	*	*	*	8/9
Castro et al. [3]	2014	*	0	*	*	*	0	*	*	*	7/9
Gunasekara et al. [4]	2018	0	*	*	*	*	0	*	*	*	7/9
Islam et al. [5]	2014	*	*	*	*	*	0	*	*	*	8/9
Leong et al. [6]	2021	*	*	*	*	*	0	*	*	*	8/9
Miller et al. [7]	2019	*	*	*	*	*	0	*	*	*	8/9
Motswagole et al. [8]	2015	*	*	*	*	*	0	*	*	0	7/9
Mulol et al. [9]	2016	*	*	*	*	*	0	*	*	*	8/9
Mulol et al. [10]	2020	*	0	*	*	*	0	*	*	0	6/9
Murhima’Alika et al. [11]	2021	*	*	*	*	*	0	*	*	*	8/9
Oiye et al. [12]	2023	*	*	*	*	*	0	*	*	*	8/9
Orr-Ewing et al. [13]	1986	*	*	*	*	*	0	*	*	*	8/9
Rabi et al. [14]	2021	0	*	*	*	*	0	*	*	*	7/9
Samuel et al. [15]	2012	*	*	*	*	*	0	*	*	*	8/9
Samuel et al. [16]	2014	*	*	*	*	*	0	*	*	*	8/9
Tongchom et al. [17]	2020	*	*	*	*	*	0	*	*	*	8/9

The symbol * equals 1 point on the NOS scale.

Table S3: Adapted Newcastle-Ottowa scale (NOS) used for quality assessment of cross-sectional studies [1]

**NOS scale**		**Selection**			**Comparability**		**Outcome**		
Studies first author	Year	“Representativeness of the sample”	“Non-respondents”	“Ascertainment of the exposure (risk factor)”	“The subjects in different outcome groups are comparable, based on the study design or analysis. Confounding factors are controlled.”		“Assessment of outcome”	"Statistical test”	Score
Agne-Djigo et al. [18]	2012	*	0	*	*	0	*	*	5/7
Agne-Djigo et al. [19]	2013	*	0	*	*	0	*	*	5/7
Bandara et al. [20]	2015	*	0	*	*	0	*	*	5/7
Bansa et al. [21]	2017	*	0	*	*	0	*	*	5/7
Butte et al. [22]	1988	0	0	*	*	0	*	*	4/7
Butte et al. [23]	1992	*	0	*	*	0	*	*	5/7
Caire et al. [24]	2002	0	0	*	*	0	*	*	4/7
Cissé et al. [25]	2002a	0	0	*	*	0	*	*	4/7
Coward et al. [26]	1982	0	0	*	*	0	*	*	4/7
Daniels et al. [27]	2019	*	0	*	*	0	*	*	5/7
Diana et al. [28]	2019	*	0	*	*	0	*	*	5/7
Diongue et al. [29]	2023	*	0	*	*	0	*	*	5/7
Ettyang et al. [30]	2005	*	*	*	*	0	*	*	6/7
Haisma et al. [31]	2003	*	*	*	*	0	*	*	6/7
Haisma et al. [32]	2005	*	0	*	*	0	*	*	5/7
Haisma et al. [33]	2006	*	0	*	*	0	*	*	5/7
Kumwenda et al. [34]	2016	*	*	*	*	0	*	*	6/7
Limon-Miro et al. [35]	2017	*	0	*	*	*	*	*	6/7
Lopez-Teros et al. [36]	2017	*	0	*	*	0	*	*	5/7
Matsiko et al. [37]	2020	*	0	*	*	0	*	*	5/7
Mazariegos et al. [38]	2016	*	*	*	*	0	*	*	6/7
Medoua et al. [39]	2012	*	0	*	*	0	*	*	5/7

Olga et al. [40]	2022	*	0	*	*	0	*	*	5/7
Rahman et al. [41]	2016	*	*	*	*	0	*	*	6/7
Romulus-Nieuwelink et al. [42]	2011	*	0	*	*	0	*	*	5/7
Rosetta et al. [43]	2005	*	*	*	*	0	*	*	6/7
Sian et al. [44]	2002	0	0	*	*	0	*	*	4/7
Urteaga et al. [45]	2018	*	0	*	*	0	*	*	5/7
Villalpando et al. [46]	1992	0	0	*	*	0	*	*	4/7
Vio et al. [47]	1991	*	*	*	*	0	*	*	6/7
Whyte et al. [48]	2022	*	0	*	*	0	*	*	5/7
Winarno et al. [49]	2019	*	0	*	*	0	*	*	5/7
Young et al. [50]	2023	*	0	*	*	*	*	*	6/7

The symbol * equals 1 point on the NOS scale.

Table S4: The Risk of Bias-2 (ROB2) tool for the quality assessment of randomized trials [51]

**ROB2 tool**		**Domain 1**	**Domain 2**	**Domain 3**	**Domain 4**	**Domain 5**	
Studies first author	Year	“Risk of bias arising from the randomization process”	“Risk of bias due to deviations from the intended interventions”	“Missing outcome data”	“Risk of bias in measurement of the outcome”	“Risk of bias in selection of the reported result”	Total
Albernaz et al. [52]	2003	low	low	low	low	low	low
Braga et al. [53]	2015	low	low	low	low	low	low
Cissé et al. [54]	2002b	low	low	low	low	low	low
Galpin et al. [55]	2007	low	low	low	low	low	low
Kumwenda et al. [56]	2014	low	low	low	low	low	low
Lokonon et al. [57]	2020	low	low	low	low	low	low
Mofid et al. [58]	2021	low	low	low	low	low	low
Moore et al. [59]	2007	low	low	low	low	low	low
Owino et al. [60]	2007	low	low	low	low	low	low
Owino et al. [61]	2011	low	low	low	low	low	low
Wells et al. [62]	2012	low	low	low	low	low	low

low: risk of bias low - some concerns - high: risk of bias high

Table S5: Formula used pooling of means and standard deviations [63, 64]

**Pooling means**	**Pooling standard deviations**
N1: sample size cohort 1<br>*N2*: sample size cohort 2<br><br>*M1*: mean cohort 1<br>*M2*: mean cohort 2<br>*M1,2*: pooled mean cohort 1 and cohort 2	*N1* - 1: sample size cohort 1 minus one<br>*N2* - 1: sample size cohort 2 minus one<br>Number of cohorts: here 2<br><br>*SD1*: standard deviation cohort 1<br>*SD2*: standard deviation cohort 2<br>*SD1,2*: pooled standard deviation cohort 1 and 2
$M1{,}2 = \frac{N1 * M1 + N2 * M2}{N1 + N2} \ldots$	$SD1{,}2 = \sqrt{\frac{(N1-1)* (SD1)^2 + (N2-1)*(SD2)^2}{N1+N2-2}} \ldots$
This formula can be expanded dependent on the number of cohorts by using the same methodology.	

Table S6: Data extraction and synthesis

Reference	Country of study	Maternal age (years)	Maternal weight (kg)	Mother-infant characteristics or interventions	Infantile age (months)	Infantile weight (kg)	n value	Human milk volume intake (ml/day)	Human milk volume intake (m/kg/day)	
Agne-Dijgo et al. 2012 [18]	*Senegal*	*28.1, SD 4*	*64, SD 9.1*	*Maternal vitamine A supplement*	*6.1, SD 0.4*	*7.6, SD1*	*13*	*≈ 900, SD 100*	*≈ 118.4, SD 20.4*	
		29.4, SD 6.7	*58.8, SD 12.2*	*Controle group*	*6.4, SD 0.4*	*7.3, SD 1*	*19*	*≈ 800, SD 100*	*≈ 109.6, SD 20.3*	
Agne-Dijgo et al. 2013 [19]	Senegal	28.73, SD 5.3	64.5, SD 9.5	Exclusively breastfed infants	5.56, SD 0.49	7.13, SD 0.8	15	≈ 1020, SD 140	≈ 143.1, SD 25.4	
		28.97, SD 5.7	60.52, sd 10.4	Partially breastfed infants	5.7, SD 0.44	7.48, SD 1	44	≈ 850, SD 230	≈ 113.6, SD 34.3	
Albernaz et al. 2003 [52]	Brazil			Lactation counseling	≈ 3.75	≈ 6.31, SD 0.6	37	761, SD 184	≈ 120.6, SD 31.7	X
				Controle group		≈ 6.46, SD 0.8	31	723, SD 241	≈ 111.9, SD 40	X
Bandara et al. 2015 [20]	Sri Lanka	27.8, SD 6.2	51.2, SD 8.6		≈ 1	≈ 3.9, SD 0.6	16	≈ 652.4, SD 123.3	≈ 172, SD 26	
			55.9, SD 9.9		≈ 3	≈ 5.4, SD 0.5	16	≈ 750.5, SD 212.6	≈ 143, SD 32	
			46.9, SD 12.9		≈ 5	≈ 6.5, SD 1	16	≈ 778.6, SD 151.5	≈ 123, SD 23	
Bansa et al. 2017 [21]	Ghana	≈ 27.5, SD 6			3		114	≈ 680.6, SD 581.6		
Braga et al. 2015 [53]	Brazil	27.2, SD 4.2	64.9, SD 8.7	Maternal hormanal implant	≈ 0.25	≈ 3.1, SD 0.4	12	343.6, SD 102.5	≈ 110.8, SD 35.4	
					≈ 1.25	≈ 4.8, SD 0.5	12	775, SD 277.6	≈ 161.1, SD 60.4	
		26.3, SD 3.1	70.1, SD 11	Controle group	≈ 0.25	≈ 3.2, SD 0.4	11	388.2, SD 170.4	≈ 123.2, SD 56.4	X
					≈ 1.25	≈ 4.8, SD 0.7	10	815.4, SD 184.1	≈ 169.9, SD 44.7	X
Butte et al. 1992 [23]	Mexico				4.39, SD 0.27	6.2, SD 0.8	15	≈ 859.2, SD 140.8	≈ 139.3, SD 29.1	
					6.26, SD 0.24	6.8, SD 0.9	15	≈ 843.7, SD 145.6	≈ 123.5, SD 26.8	
Butte et al. 1988 [22]	USA	27, SD 3	61.6, SD 7.9		≈ 3.32, SD 1.38	6.3, SD 1.5	9	≈ 629.1, SD 61.1	≈ 99.4, SD 25.6	
Caire et al. 2002 [24]	Mexico	21.7, SD 3.4	62.7, SD 8.1		≈ 1.5	4.5, SD 0.9	10	≈ 605.8, SD 287.4	≈ 134.6, SD 69.3	
Castro et al. 2014 [3]	Chile	24	70	Polluted area, exclusively breastfed infants	≈ 2.5	≈ 6.3		≈ 840, SD 300	≈ 134	X
				Polluted area, non-exclusively breastfed infants				≈ 750, SD 200	≈ 119.6	X
				Polluted area, exclusively breastfed infants	≈ 5.9	≈ 8.2		≈ 1000, SD 400	≈ 122.4	X
				Polluted area, non-exclusively breastfed infants				≈ 820, SD 200	≈ 100.4	X
		29	69	Controle, exclusively breastfed infants	≈ 1.99	≈ 5.3		≈ 960, SD 200	≈ 181.5	X
				Controle, non-exclusively breastfed infants				≈ 560, SD 300	≈ 105.9	X
				Controle, exclusively breastfed infants	≈ 5.1	≈ 7.2		≈ 870, SD 400	≈ 121.5	X
				Controle, non-exclusively breastfed infants				≈ 670, SD 400	≈ 93.6	X
Cissé et al. 2002a [25]	Senegal	24, SD 4			3.7		11	≈ 866, SD 104.9		

Cissé et al. 2002b [54]	Senegal	27, SD 6	≈ 58	Infantile millet supplemented	3		41	≈ 891.3, SD 180.6		
			≈ 59	Infantile maize supplemented			35	≈ 963.1, SD 199		
			≈ 56	Non-supplemented infants			57	≈ 915.5, SD 201		
Coward et al. 1982 [26]	Gambia				≈ 2	≈ 5	4	752, SD 18	152, SD 9	
					≈ 7	≈ 7.5	4	757, SD 22	101, SD 7	
	Papua New Guinea				≈ 2	≈ 4.8	17	670, SD 46	140, SD 7	
					≈ 7	≈ 6.5	8	936, SD 61	145, SD 7	
Daniels et al. 2019 [27]	Indonesia	25.8, SD 6.1	54.5, SD 9.6		3.3, SD 0.8		110	787, SD 148	≈ 132	
Diana et al. 2019 [28]	*Indonesia*	*25.8, SD 6.1*			*3.3, SD 0.8*		*112*	*787, SD 149*		
Diongue et al. 2023 [29]	Senegal			Exclusively breastfed infants	4.9, SD 0.6		46	≈ 1008.7, SD 187.4		
				Non-exclusively breastfed infants	4.9, SD 0.5		94	≈ 888.4, SD 204.9		
Ettyang et al. 2005 [30]	Kenya		49.5, SD 0.8		3.69, SD 1.26	5, SD 0.9	10	552, SD 22	115, SD 5	
Galpin et al. 2007 [55]	Malawi	23, SD 5	50.8, SD 5.4	Before complimentary food 25 g/day to infant	5.3, SD 0.3	7, SD 0.7	15	≈ 927.2, SD 183.5	≈ 128.2, SD 20.4	
		26, SD 4	55.2, SD 5.8	Before complimentary food 50 g/day to infant	5.5, SD 0.4	7, SD 0.9	14	≈ 894.2, SD 160.2	≈ 123.3, SD 18.5	
		28, SD 4	50.7, SD 4.4	Before complimentary food 72 g/day to infant		6.5, SD 0.3	12	≈ 814.6, SD 105.8	≈ 124.3, SD 14.6	
		23, SD 5	50.8, SD 5.4	After complimentary food 25 g/day to infant	≈ 7.05	7.3, SD 0.6	15	≈ 875.7, SD 187.4	≈ 111.7, SD 20.4	
		26, SD 4	55.2, SD 5.8	After complimentary food 50 g/day to infant	≈ 7.25	7.5, SD 0.9	14	≈ 903.9, SD 181.6	≈ 114.6, SD 17.5	
		28, SD 4	50.7, SD 4.4	After complimentary food 72 g/day to infant		6.9, SD 0.4	12	≈ 773.8, SD 115.6	≈ 107.8, SD 13.6	
Gunasekara et al. 2018 [4]	Sri Lanka	29.8, SD 4	≈ 54.2		3	5.3, SD 0.7	30	≈ 747.6, SD 135.9	≈ 141.1, SD 31.7	
			≈ 53.7		6	6.5, SD 0.7	30	≈ 779.6, SD 165	≈ 119.9, SD 28.5	
Haisma et al. 2003 [31]	Brazil	30, SD 5	62.7, SD 9.3	Exclusively breastfed infants	4	6.6, SD 0.9	35	≈ 806, SD 154.3	≈ 122.1, SD 28.7	
		24.6, SD 6.1	58.3, SD 6.9	Predominantly breastfed infants	4	6.4, SD 0.6	16	≈ 778, SD 120.1	≈ 121.6, SD 22	
		28.5, SD 5.6	65.7, SD 11.4	Partially breastfed infants	4	6.5, SD 0.6	19	≈ 603, SD 270.8	≈ 92.8, SD 42.5	
Haisma et al. 2006 [33]	*Brazil*			*Middle socioeconomic status*	*8*	*8.7, SD 1*	*32*	*689, SD 334*	*≈ 79.2 SD 39.5*	
				Low socioeconomic status	*8*	*8.2, SD 1.1*	*33*	*638, SD 325*	*≈ 77.8, SD 41*	
Haisma et al. 2005 [32]	Brazil			Exclusively breast milk	8.7	8.4, SD 0.9	32	761, SD 231	≈ 90.6, SD 29.2	X
				Complimentary cow's milk	8.7	8.7, SD 1.2	26	464, SD 352	≈ 53.3, SD 41.1	X
Islam et al. 2014 [5]	Bangladesh	25.7, SD 3.8	47.6, SD 7.2		≈ 1	3.8, SD 0.3	20	≈ 698.1, SD 221.4	≈ 180.6, SD 46.6	
			46.4, SD 7.4		≈ 3	5.3, SD 0.5	19	≈ 749.5, SD 151.5	≈ 141.7, SD 22.3	X
			45.6, SD 7.3		≈ 6	6.5, SD 0.7	20	≈ 819.4, SD 132	≈ 125.2, SD 14.6	

Kumwenda et al. 2014 [56]	Malawi	26, SD 6.2	52.1, SD 6.7	Controle group	9.9, SD 0.5	8, SD 1.1	79	≈ 708.7, SD 219	≈ 88.6, SD 30	
		26.3, SD 7.7	52.4, SD 9	10 g/day complimentary feeding to infant		8.1, SD 1	75	≈ 768.9, SD 271.6	≈ 94.9, SD 35.5	
		26, SD 6.1	52.7, SD 7.8	20 g/day complimentary feeding to infant	9.8, SD 0.5	8.1, SD 1.1	98	≈ 737.9, SD 232.2	≈ 91.1, SD 31.2	
		27.2, SD 6.2	53.3, SD 8.6	40 g/day complimentary feeding to infant		8, SD 1	107	≈ 710.7, SD 242.2	≈ 88.8, 32.3	
Kumwenda et al. 2016 [34]	*Malawi*	*26*	*52.7, SD 8.1*		*≈ 9.5*	*8.1, SD 1*	*358*	*≈ 730.1, SD 236.9*	*≈ 90.1, SD 31.3*	
Limon-Miro et al. 2017 [35]	Mexico	22.1, SD 1	62.1, SD 13	Rural setting	3, SD 1.6	6.1, SD 1.4	30	799, SD 193	≈ 131, SD 43.6	
		27.5, SD 1	64.4, SD 11	Urban setting	3.6, SD 1.6	6.5, SD 0.9	32	707, SD 201	≈ 108.8, SD 34.4	
Lokonon et al. 2020 [57]	Benin	27.2, SD 5.6		Educational intervention breastfeeding	4.57, SD 0.67	6.8, SD 1	53	≈ 874, SD 148.1	≈ 128.5, SD 28.8	
		27.7, SD 6.9		Controle group	5.05, SD 0.35	6.7, SD 1	50	≈ 817.7, SD 183.1	≈ 122, SD 32.8	
Lopez-Teros et al. 2017 [36]	Mexico	27.8, SD 6	63, SD 11.5	Urban setting	3.6, SD 1.5	6.4, SD 0.9	26	710, SD 169	≈ 110.9, SD 30.7	
		22.4, SD 7	70, SD 14	Rural setting	3.1, SD 1.6	6.1, SD 1.4	30	800, SD 190	≈ 131.1, SD 43.3	
Matsiko et al. 2020 [37]	The Netherlands	30.8, SD 0.8	63.8, SD 13.2	Mother-infant pairs from the Netherlands	3.4, SD 1	≈ 6.2, SD 0.6	5	≈ 738.1, SD 63.7	≈ 119, SD 15.4	
	Rwanda	30.5, SD 6.1	60.2, SD 5.3	Mother-infant pairs from Rwanda	3.7, SD 0.6	≈ 6.7, SD 1.4	8	≈ 875.3, SD 253.6	≈ 130.6, SD 46.2	
Mazariegos et al. 2016 [38]	Guatemala	26.8, SD 5.3	≈ 60.3	Exclusively breastfed infants	3.6, SD 0.4	6.5, SD 0.9	18	≈ 762.8, SD 102	≈ 117.4, SD 22.6	
		27, SD 6.6	≈ 59.9	Non-exclusively breastfed infants	3.5, SD 0.5	6.3, SD 0.8	18	≈ 603.1, SD 190.2	≈ 95.7, SD 32.5	
Medoua et al. 2012 [39]	Cameroon	26.6, SD 5.1	69.2, SD 11.4		2.7, SD 1.3	6.1, SD 1.4	44	700.8, SD 204.3	≈ 114.9, SD 42.6	
Miller et al. 2019 [7]	Kenya	≈ 27, SD 6.3			≈ 1.5		119	≈ 700.3		
					≈ 6		119	≈ 933.1		
Mofid et al. 2021 [58]	Peru	24.4, SD 6.7		Maternal postnatal Albendazole	1		109	≈ 756, SD 167		
					6		107	≈ 903, SD 165.5		
		26.4, SD 7.8		Placebo group	1		90	≈ 774, SD 170.8		
					6		93	≈ 908, SD 173.6		
Moore et al. 2007 [59]	Bangladesh	28.1, SD 5.2	45, SD 4.4	Lactation counseling	≈ 3.3, SD 0.31	5.5, SD 0.8	44	866	≈ 157.5	
		26.9, SD 5.6	44.7, SD 7.3	No lactation counseling	≈ 3.26,SD0.34	5.7, SD 0.7	54	860	≈ 150.1	
Motswagole et al. 2015 [8]	Botswana		62.1, SD 11.1		≈ 1.5		50	611.2, SD 188.1		
			62.6, SD 12.4		3	6.1, SD 1	31	791.3, SD 283.4	≈ 129.7, SD 50.7	X
			62.5, SD 13.8		6	7.6, SD 1.3	23	838.1, SD 248.1	≈ 110.3, SD 37.6	X
Mulol et al. 2016 [9]	South Africa			HIV positive mother	≈ 1.5	4.8, SD 0.6	21	≈ 806.8, SD 179.6	≈ 166.7, SD 42	
				HIV negative mother		4.9, SD 0.6	24	≈ 920.4, SD 216.5	≈ 189, SD 50.9	
				HIV positive mother	3	6.1, SD 0.7	28	≈ 872.8, SD 182.5	≈ 142.9, SD 34.3	
				HIV negative mother		6.5, SD 1	45	≈ 898.1, SD 220.4	≈ 139.2, SD 39.9	
				HIV positive mother	6	7.4, SD 0.9	27	≈ 845.6, SD 284.5	≈ 115.1, SD 41	
				HIV negative mother		7.9, SD 1.3	45	≈ 875.7, SD 277.7	≈ 111.3, SD 40	
				HIV positive mother	9	8.4, SD 1.2	24	≈ 659.2, SD 272.8	≈ 78.9, SD 34.4	

				HIV negative mother		9.1, SD 1.5	43	≈ 724.3, SD 255.3	≈ 79.9, SD31.2	
				HIV positive mother	12	9.3, SD 0.8	13	≈ 733, SD 278.6	≈ 78.7, SD 30.7	
				HIV negative mother		9.7, SD 1.4	33	≈ 692.2, SD 256.3	≈ 71.2, SD 28.3	
Mulol et al. 2020 [10]	Benin	27.2, SD 6			3	5.5, SD 1.4	127	≈ 640.9, SD 204	≈ 116.5, SD 47.5	X
	Central African Republic	28.7, SD 4.8			3	5.9, SD 0.9	46	≈ 711.9, SD 177.6	≈ 120.7, SD 35.3	
					6	7.2, SD 0.9	35	≈ 698.1, SD 193.7	≈ 97, SD 29.5	
	Morocco	30.2, SD 5.6			3	6.4, SD 1	68	≈ 488.6, SD 127.1	≈ 76.4, SD 23.2	
					6	8.1, SD 1.1	50	≈ 543.9, SD 137.2	≈ 67.1, SD 19.2	X
	South Africa	24.9, SD 5.4			3	6.6, SD 1	74	≈ 887.6, SD 204.5	≈ 134.5, SD 37.1	X
					6	7.9, SD 1.3	72	≈ 864.3, SD 278.4	≈ 109.4, SD 39.6	X
	Tanzania	27.8, SD 5.6			3	6.1, SD 1.2	40	≈ 684.7, SD 201.6	≈ 112.2, SD 39.7	X
					6	7.5, SD 1.5	36	≈ 558.4 SD 253.3	≈ 74.5, SD 36.9	X
Murhima'Alika et al. 2021 [11]	Democratic Republic of Congo	24.4, SD 5.1	55.2, SD 7.5	Mothers with malnutrition during infancy	5.4, SD 2.3	6.7, SD 1.3	39	≈ 809.4, SD 147.8	≈ 120.8, SD 32.2	
		26, SD 6.1	55.9, SD 7	Mothers without malnutrition during infancy		6.6, SD 1.4	40	≈ 803.3, SD 166.4	≈ 121.7, SD 36.1	
Oiye et al. 2023 [12]	Kenya	28.8, SD 6.2	58.2, SD 6	HIV positive mother	≈ 1.5	4.7, SD 0.6	68	≈ 700, SD 107.8	≈ 148.9, SD 29.8	
		25.5, SD 6.3	58.6, SD 6	HIV negative mother		4.8, SD 0.7	65	≈ 698.1, SD 117.5	≈ 145.4, SD 32.4	
				HIV positive mother	6		60	≈ 932, SD 117.5		
				HIV negatief mother			62	≈ 935, SD 103.9		
Olga et al. 2022 [40]	UK	33.6, SD 4.3			≈ 1.25	5.1, SD 0.7	70	≈ 780, SD 160	≈ 150, SD 20	
Orr-Ewing et al. 1986 [13]	Papua New Guinea				≈ 0.75		16	≈ 588.4, SD 97.1		
					≈ 0.75		1	≈ 611.7		
					≈ 2.3		21	≈ 712.6, SD 159.2		
							2	≈ 759.2		
					≈ 3.55		20	≈ 789.3, SD 147.6		
							1	≈ 987.4		
					≈ 5.05		22	≈ 837.9, SD 152.4		
					≈ 7.05		21	≈ 868, SD 194.2		
							2	≈ 953.4		
					≈ 9.3		18	≈ 874.8, SD 204.9		
							1	≈ 886.4		
					≈ 11.8		20	≈ 786.4, SD 164.1		
							4	≈ 724.3		
Owino et al. 2011 [61]	Democratic Republic of Congo		51.4, SD 4	Complemenatry feeding (UNIMIX)	≈ 9.5	7.6, SD 1	29	≈ 658.3, SD 277.1	≈ 87.8, SD 35.7	
			52.7, SD 6.6	Complemenatry feeding (RUCF)		8, SD 1	29	≈ 684.5, SD 229.2	≈ 86.5, SD 29.1	

Owino et al. 2007 [60]	Zambia	25.6, SD 5.3	58.5, SD 9.6	Controle group	9	8.6, SD 1.1	27	≈ 634, SD 214.6	≈ 73.7, SD 26.7	X
		27.2, SD 5.9	60.3, SD 14.5	Complemenatry feeding (CBM)		9, SD 1.5	12	≈ 596.1, SD 263.1	≈ 66.2, SD 31.2	X
		27.2, SD 4.7	60.1, SD 14.4	Complemenatry feeding (CBMA)		8.9, SD 1.4	14	≈ 616.5, SD 187.4	≈ 69.3, SD 23.7	X
Rabi et al. 2021 [14]	Marroco		73.8, SD 11.8		1	4.7, SD 0.5	70	≈ 425, SD 107.5	≈ 90.4, SD 24.8	
			66.6, SD 11.9		3	6.4, SD 1	68	≈ 568.5, SD 141.8	≈ 88.8, SD 26.1	
			64.9, SD 11.6		6	8.1, SD 1.1	50	≈ 586.6, SD 138.4	≈ 72.4, SD 19.7	
			64, SD 12.1		9	8.8, SD 0.9	42	≈ 497.3, SD 137.2	≈ 56.5, SD 16.6	
			62.3, SD 12.1		12	9.5, SD 1	34	≈ 422.8, SD 97.8	≈ 44.5, SD 11.3	
Rahman et al. 2016 [41]	Pakistan	28.1, SD 6.1	56.7, SD 12.8	Depressed mothers	4		24		89.3, SD 38.1	
		27.5, SD 6	54.5, SD 10.2	Non-depressed mothers			31		83.9, SD 29	
Romulus-Nieuwelink et al. 2011 [42]	Brazil				8	≈ 8.4, SD 1	65	655, SD 336	≈ 77.8, SD 41	
Rosetta et al. 2005 [43]	Bangladesh		38.3, SD 4.3		≈ 12.2	7.2, SD 1.1	17	672, SD 180.4	≈ 93.5, SD 28.6	
			35.6, SD3.9		≈ 12.04	7, SD 1	12	749.3, SD 189	≈ 107.5, SD 31.3	
Samuel et al. 2012 [15]	India	23, SD 2.9	57.3, SD 11.6		≈ 0.98	3.8, SD 0.6	50	627, SD 170	166, SD 38	
			56.3, SD 11.5		≈ 3.09	5.6, SD 0.8	48	744, SD 183	132, SD 29	
			56.7, SD 12.4		≈ 5.98	7.2, SD 1	49	608, SD 235	87, SD 34	X
Samuel et al. 2014 [16]	*India*	*23, SD 2.9*	*60.4, SD 12.1*		*≈ 0.98*	*3.8, SD 0.6*	*50*	*627, SD 170*	*≈ 165, SD 51.8*	
					≈ 3.09	*5.6, SD 0.8*	*48*	*744, SD 183*	*≈ 132, SD 37.8*	
					≈ 5.98	*7.2, SD 1*	*49*	*608, SD 235*	*≈ 84.4, SD 34.7*	*x*
Sian et al. 2002 [44]	China	23, SD 2	60, SD 7		≈ 1.58, SD0.36	5.4, SD 0.8	18	≈ 850, SD 210	≈ 158.9, SD 46	
Tongchom et al. 2020 [17]	Thailand			Exclusively breastfed infants	≈ 1.5		29	≈ 721.5, SD 169.9		
				Predominantly breastfed infants			37	≈ 737, SD 193.3		
				Partially breastfed infants			37	≈ 561.2, SD 290.7		
				Exclusively breastfed infants	3		26	≈ 753.6, SD 138		
				Predominantly breastfed infants			36	≈ 741.8, SD 171.4		
				Partially breastfed infants			32	≈ 538, SD 312.6		
				Exclusively breastfed infants	6		16	≈ 726.3, SD 175.1		
				Predominantly breastfed infants			11	≈ 837.8, SD 133		
				Partially breastfed infants			58	≈ 632, SD 178.1		
Urteaga et al. 2018 [45]	Bolivia	28.8, SD 5.9	57.3, SD 5.2		2.2, SD 0.4	6, SD 0.4	9	899.3, SD 160.9	≈ 149.9, SD 28.6	
			57.4, SD 15.9		5, SD 0.8	7, SD 1.1	9	876.7, SD 146.2	≈ 125.2, SD 28.7	
Villalpando et al. 1992 [46]	Mexico	26, SD 6.1	51.8, SD 5.3		4		15	≈ 859.2, SD 141.7		
			48.8, SD 6.3		6		15	≈ 843.7, SD 145.6		

Vio et al. 1991 [47]	Chile	28, SD 3.5	57.5, SD 6	Smoking mothers (≥ 4 cigararettes/day)	≈1.35,SD 0.22	4.3, SD 0.4	10	≈ 672.8, SD 106.8	≈ 157.6, SD 29.8
		27, SD 4	54.1, SD 4.1	Non-smoking mothers	≈1.71,SD 0.46	4.5, SD 0.7	10	≈ 933, SD 116.5	≈ 205.5, SD 41.1
Wells et al. 2012 [62]	Iceland	29.7, SD 4.4	73, SD 15.5	Complementary feeding	≈ 6.13, SD 0.23	8, SD 1.1	50	≈ 794.2, SD 161.2	≈ 99.8, SD 24.2
		30.7, SD 5.1	71.3, SD 12.4	Exclusively breastfed infants	≈ 6.15, SD 0.22	8, SD 1	50	≈ 874.8, SD 153.4	≈ 109.2, SD 23.8
Whyte et al. 2022 [48]	Jamaica	30.4, SD 6.7	64.8, SD 13.4	Rural, exclusively breastfed infants	1.6	5.1, SD 0.6	17	≈ 1045.1, SD 202.6	≈ 205, SD 29.4
		27.1, SD 6.1	66.4, SD 15.1	Rural, non-exclusively breastfed infants	1.6	5.2, SD 1.2	9	≈ 948.4, SD 233.4	≈ 184.4, SD 33.3
		29.2, SD 5.4	76.3, SD 22.7	Urban, exclusively breastfed infants	1.8	5.5, SD 0.7	13	≈ 928.2, SD 295.2	≈ 168.8, SD 45.7
		28, SD 6.4	65.6, SD 12	Urban, non exclusively breastfed infants	1.7	5.1, SD 0.7	22	≈ 683.8, SD 339	≈ 132.5, SD 81.7
Winarno et al. 2019 [49]	Indonesia	30.8, SD 5.2	59.2, SD 10.9		3	≈ 6, SD 0.7	30	≈ 761.2, SD 113.6	≈ 130, SD 20.2
			59.1, SD 11.4		6	≈ 7.1, SD 0.8	30	≈ 764.1, SD 174.8	≈ 108.5, SD 25.2
Young et al. 2023 [50]	India	24.7, SD 3.9	50.2, SD 9.2	Underweight mothers	3		34	756, SD 182	
				Normal weight mothers	3		160	717, SD 189	
				Overweight/obese mother	3		38	726, SD 163	
**No further data processing allowed as human milk volume intake was reported in medians and IQR.**									
**Reference**	**Country of study**	**Median maternal age (years)**	**Median maternal weight (kg)**	**Mother-infant characteristics or interventions**	**Infantile age (months)**	**Mean infantile weight (kg)**	**n value**	**Median human milk volume intake**	**Mean human milk volume intake**
Caire-Juvera et al. 2012 [2]	Mexico	16.8, IQR 15.8-17.1	50.2, IQR 49.5-58.1	Adolecent mothers	1		21	660, IQR 530-810 g/day	/
			49.5, IQR 47.1-59		3			610, IQR 420-845 g/day	
		23.4, IQR 22.2-26.5	57.6, IQR 53.7-64.3	Adult mothers	1		20	810, IQR 680-920 g/day	
			56.9, IQR 52.1-65.1		3			815, IQR 635-1,115 g/day	
Leong et al. 2021 [6]	Indonesia			Exclusively breastfed infants	2		133	771, IQR 664-882 ml/day	/
					5	6.7, SD 0.8	63	799, IQR 716-895 ml/day	
				Partially breastfed infants	2	4.8, SD 0.7	26	450, IQR 163-599 ml/day	
					5		96	631, IQR 520-737 ml/day	

n value equaling the number of mother-infant pairs.
Values reported as mean, SD (or median, inter quartile range (IQR) in last two studies)
Italic data were merged with data from adjacent articles.
(≈) symbol depicting synthesized values after extraction.
(X) symbol depicting a mismatch between sample sizes of infantile weight and human milk volume intake dat

Refenences

1. Kenniscentrum voor de Gezondheidszorg Gent. Newcastle - Ottawa quality assessment scale [Available from: https://www.kcgg.ugent.be/pdf/NEWCASTLE-OTTAWA_QUALITY_ASSESSMENT_SCALE.pdf.
2. Caire-Juvera G, Casanueva E, Bolaños-Villar AV, De Regil LM, De la Barca AMC. No changes in weight and body fat in lactating adolescent and adult women from Mexico. American Journal of Human Biology. 2012;24(4):425-31.
3. Castro F, Harari F, Llanos M, Vahter M, Ronco AM. Maternal-Child Transfer of Essential and Toxic Elements through Breast Milk in a Mine-Waste Polluted Area. American Journal of Perinatology. 2014.
4. Gunasekara P, Asha Lakmali GA, Amarasena S, Hettiarachchi M. Exclusive breastfeeding up to six months: Are we getting the right figures? SriLanka J Child Health. 2018;47(4):311-5.
5. Islam MM, Brown KH. Zinc transferred through breast milk does not differ between appropriate- and small-for-gestational-age, predominantly breast-fed Bangladeshi infants. Journal of Nutrition. 2014;144(5):771-6.
6. Leong C, Gibson RS, Diana A, Haszard JJ, Rahmannia S, Ansari MB, et al. Differences in Micronutrient Intakes of Exclusive and Partially Breastfed Indonesian Infants from Resource-Poor Households are Not Accompanied by Differences in Micronutrient Status, Morbidity, or Growth. Journal of Nutrition. 2021;151(3):705-15.
7. Miller JD, Young SL, Boateng GO, Oiye S, Owino V. Greater household food insecurity is associated with lower breast milk intake among infants in western Kenya. Maternal and Child Nutrition. 2019;15(4).
8. Motswagole BS, Matenge STP, Mongwaketse T, Bogopa J, Kobue-Lekalake R, Mosetlha K, et al. Application of the deuterium-oxide dose-to-mother technique to determine the exclusivity of breastfeeding in women in Kanye, Botswana. South African Journal of Clinical Nutrition. 2015;28(3):128-33.
9. Mulol H, Coutsoudis A. Breastmilk Output in a Disadvantaged Community with High HIV Prevalence as Determined by the Deuterium Oxide Dose-to-Mother Technique. Breastfeeding Medicine. 2016;11(2):64-9.
10. Mulol H, Coutsoudis A, Hounkpatin WA, Urio E, Wabolou PK, Sissinto Y, et al. Is exclusive breastfeeding an option or a necessity in africa? A pooled study using the deuterium oxide dose-to-mother technique. Journal of Public Health in Africa. 2020;11(1).
11. Murhima'Alika CC, Balemba GM, Lyabayungu PMB, Mulume'oderhwa GM, Munthali G, Owino V, et al. Human Milk output among mothers previously treated for severe acute malnutrition in childhood in Democratic Republic of Congo. BMC Nutrition. 2021;7(1).
12. Oiye S, Mwanda W, Filteau S, Owino V. HIV-Infected and HIV-Uninfected Western Kenyan Women Produce Equivalent Amounts of Breast Milk at 6 Wk and 6 Mo Postpartum: A Prospective Cohort Study Using Deuterium Oxide Dose-to-Mother Technique. The Journal of nutrition. 2023;153(1):27-33.
13. Orr-Ewing AK, Heywood PF, Coward WA. Longitudinal measurements of breast milk output by a 2H2O tracer technique in rural Papua New Guinean women. Human Nutrition: Clinical Nutrition. 1986;40(6):451-67.
14. Rabi B, Benjeddou K, Idrissi M, Rami A, Mekkaoui B, Hamdouchi AE, et al. Effects of breastfeeding on maternal body composition in moroccan lactating women during

twelve months after birth using stable isotopic dilution technique. Nutrients. 2021;13(1):1-14.
15. Samuel TM, Thomas T, Bhat S, Kurpad AV. Are infants born in baby-friendly hospitals being exclusively breastfed until 6 months of age. European Journal of Clinical Nutrition. 2012;66(4):459-65.
16. Samuel TM, Thomas T, Thankachan P, Bhat S, Virtanen SM, Kurpad AV. Breast milk zinc transfer and early post-natal growth among urban South Indian term infants using measures of breast milk volume and breast milk zinc concentrations. Maternal and Child Nutrition. 2014;10(3):398-409.
17. Tongchom W, Pongcharoen T, Judprasong K, Udomkesmalee E, Kriengsinyos W, Winichagoon P. Human Milk Intake of Thai Breastfed Infants During the First 6 Months Using the Dose-to-Mother Deuterium Dilution Method. Food and Nutrition Bulletin. 2020;41(3):343-54.
18. Agne-Djigo A, Idohou-Dossou N, Kwadjode KM, Tanumihardjo SA, Wade S. High prevalence of vitamin A deficiency is detected by the modified relative dose-response test in six-month-old Senegalese breast-fed infants. J Nutr. 2012;142(11):1991-6.
19. Agne-Djigo A, Kwadjode KM, Idohou-Dossou N, Diouf A, Guiro AT, Wade S. Energy intake from human milk covers the requirement of 6-month-old Senegalese exclusively breast-fed infants. Br J Nutr. 2013;110(10):1849-55.
20. Bandara T, Hettiarachchi M, Liyanage C, Amarasena S, Wong WW. The deuterium oxide-to-the-mother method documents adequate breast-milk intake among Sri Lankan infants. Journal of Nutrition. 2015;145(6):1325-9.
21. Bansa DK, Awua AK, Boatin R, Adom T, Brown-Appiah EC, Amewosina KK, et al. Cross-sectional assessment of infants' exposure to toxic metals through breast milk in a prospective cohort study of mining communities in Ghana. BMC Public Health. 2017;17(1):505.
22. Butte NF, Wong WW, Patterson BW, Garza C, Klein PD. Human-milk intake measured by administration of deuterium oxide to the mother: A comparison with the test-weighing technique. American Journal of Clinical Nutrition. 1988;47(5):815-21.
23. Butte NF, Villalpando S, Wong WW, Floreshuerta S, Hernandezbeltran MD, Smith EO, et al. HUMAN-MILK INTAKE AND GROWTH FALTERING OF RURAL MESOAMERINDIAN INFANTS. American Journal of Clinical Nutrition. 1992;55(6):1109-16.
24. Caire G, Calderon de la Barca AM, Bolanos AV, Valencia ME, Coward AW, Salazar G, et al. Measurement of deuterium oxide by infrared spectroscopy and isotope ratio mass spectrometry for quantifying daily milk intake in breastfed infants and maternal body fat. Food and Nutrition Bulletin. 2002;23(3 SUPP):38-41.
25. Cissé AS, Bluck L, Diaham B, Dossou N, Guiro AT, Wade S. Use of Fourier transformed infrared spectrophotometer (FTIR) for determination of breastmilk output by the deuterium dilution method among Senegalese women. Food and Nutrition Bulletin. 2002;23(3 SUPP):138-41.
26. Coward WA, Cole TJ, Sawyer MB, Prentice AM. Breast-milk intake measurement in mixed-fed infants by administration of deuterium oxide to their mothers. Hum Nutr Clin Nutr. 1982;36(2):141-8.
27. Daniels L, Gibson RS, Diana A, Haszard JJ, Rahmannia S, Luftimas DE, et al. Micronutrient intakes of lactating mothers and their association with breast milk

concentrations and micronutrient adequacy of exclusively breastfed Indonesian infants. American Journal of Clinical Nutrition. 2019;110(2):391-400.
28. Diana A, Haszard JJ, Houghton LA, Gibson RS. Breastmilk intake among exclusively breastfed Indonesian infants is negatively associated with maternal fat mass. European Journal of Clinical Nutrition. 2019;73(8):1206-8.
29. Diongue O, Diouf A, Ndour PS, Badiane A, Thiam M, Faye MH, et al. Exclusive Breastfeeding Measured by Deuterium-Oxide Turnover Method is Associated with Motor Development in Rural Senegalese Infants. Journal of Nutrition. 2023.
30. Ettyang GA, Van Marken Lichtenbelt WD, Esamai F, Saris WHM, Westerterp KR. Assessment of body composition and breast milk volume in lactating mothers in pastoral communities in Pokot, Kenya, using deuterium oxide. Annals of Nutrition and Metabolism. 2005;49(2):110-7.
31. Haisma H, Coward WA, Albernaz E, Visser GH, Wells JCK, Wright A, et al. Breast milk and energy intake in exclusively, predominantly, and partially breast-fed infants. European Journal of Clinical Nutrition. 2003;57(12):1633-42.
32. Haisma H, Wells JCK, Coward WA, Duro Filho D, Victora CG, Vonk RJ, et al. Complementary feeding with cow's milk alters sleeping metabolic rate in breast-fed infants. Journal of Nutrition. 2005;135(8):1889-95.
33. Haisma H, Coward WA, Visser GH, Vonk R, Wells JCK, Wright A, et al. Socio-economic and environmental factors influence energy utilization in Brazilian breast-fed infants. Journal of Nutrition. 2006;136(11):2945-51.
34. Kumwenda C, Hemsworth J, Phuka J, Arimond M, Ashorn U, Maleta K, et al. Factors associated with breast milk intake among 9–10-month-old Malawian infants. Maternal and Child Nutrition. 2016;12(4):778-89.
35. Limon-Miro AT, Aldana-Madrid ML, Alvarez-Hernandez G, Antunez-Roman LE, Rodriguez-Olibarria G, Valencia Juillerat ME. Breast milk intake and mother to infant pesticide transfer measured by deuterium oxide dilution in agricultural and urban areas of Mexico. Chemosphere. 2017;181:682-9.
36. Lopez-Teros V, Limon-Miro AT, Astiazaran-Garcia H, Tanumihardjo SA, Tortoledo-Ortiz O, Valencia ME. 'Dose-to-mother' deuterium oxide dilution technique: An accurate strategy to measure vitamin a intake in breastfed infants. Nutrients. 2017;9(2).
37. Matsiko E, Hulshof PJM, Van Der Velde L, Kenkhuis MF, Tuyisenge L, Melse-Boonstra A. Comparing saliva and urine samples for measuring breast milk intake with the 2H oxide dose-to-mother technique among children 2-4 months old. British Journal of Nutrition. 2020;123(2):232-40.
38. Mazariegos M, Slater C, Ramirez-Zea M. Validity of Guatemalan Mother's Self-Reported Breast-Feeding Practices of 3-Month-Old Infants. Food and Nutrition Bulletin. 2016;37(4):494-503.
39. Medoua GN, Sajo Nana EC, Ndzana ACA, Makamto CS, Etame LS, Rikong HA, et al. Breastfeeding practices of Cameroonian mothers determined by dietary recall since birth and the dose-to-the-mother deuterium-oxide turnover technique. Maternal and Child Nutrition. 2012;8(3):330-9.
40. Olga L, Vervoort J, van Diepen JA, Gross G, Petry CJ, Prentice PM, et al. Associations between breast milk intake volume, macronutrient intake, and infant growth in a longitudinal birth cohort: the Cambridge Baby Growth and Breastfeeding Study (CBGS-BF). British Journal of Nutrition. 2022.

41. Rahman A, Hafeez A, Bilal R, Sikander S, Malik A, Minhas F, et al. The impact of perinatal depression on exclusive breastfeeding: a cohort study. Maternal and Child Nutrition. 2016;12(3):452-62.
42. Romulus-Nieuwelink JJC, Doak C, Albernaz E, Victora CG, Haisma H. Breast milk and complementary food intake in Brazilian infants according to socio-economic position. International Journal of Pediatric Obesity. 2011;6(2 -2):e508-e14.
43. Rosetta L, Kurpad A, Mascie-Taylor CG, Shetty PS. Total energy expenditure (H218O), physical activity level and milk output of lactating rural Bangladeshi tea workers and nontea workers. Eur J Clin Nutr. 2005;59(5):632-8.
44. Sian L, Krebs NF, Westcott JE, Fengliang L, Tong L, Miller LV, et al. Zinc homeostasis during lactation in a population with a low zinc intake. American Journal of Clinical Nutrition. 2002;75(1):99-103.
45. Urteaga N, San Miguel JL, Aguilar AM, Muñoz M, Slater C. Nutritional status and human milk intake of exclusively breast-fed infants at high altitude in la Paz, Bolivia. British Journal of Nutrition. 2018;120(2):158-63.
46. Villalpando SF, Butte NF, Wong WW, Flores-Huerta S, Hernandez-Beltran MJ, Smith EO, et al. Lactation performance of rural Mesoamerindians. Eur J Clin Nutr. 1992;46(5):337-48.
47. Vio F, Salazar G, Infante C. Smoking during pregnancy and lactation and its effects on breast-milk volume. American Journal of Clinical Nutrition. 1991;54(6):1011-6.
48. Whyte S, McLean-Smith J, Reid M. Concordance of the Deuterium Dose to Mother Method and 24-Hour Recall to Measure Exclusive Breastfeeding at 6 Weeks Postnatally in Rural/Urban Setting in Jamaica. Maternal and child health journal. 2022;26(10):2126-36.
49. Winarno EK, Winarno H, Susanto, Fajarwati T, Thomas T. Assessment of human milk intake by breastfed infants using deuterium oxide dose-to-mother technique in "Tumbuh Kembang Anak" cohort, Indonesia. Atom Indonesia. 2019;45(1):51-8.
50. Young MF, Faerber EC, Mehta RV, Ranjan S, Shetty SA, Ramakrishnan U, et al. Maternal nutritional status and milk volume and composition in India: an observational study. American Journal of Clinical Nutrition. 2023;117(4):830-7.
51. Sterne JAC, Savović J, Page MJ, Elbers RG, Blencowe NS, Boutron I, et al. RoB 2: a revised tool for assessing risk of bias in randomised trials. Bmj. 2019;366:l4898.
52. Albernaz E, Victora CG, Haisma H, Wright A, Coward WA. Lactation counseling increases breast-feeding duration but not breast milk intake as measured by isotopic methods. Journal of Nutrition. 2003;133(1):205-10.
53. Braga GC, Ferriolli E, Quintana SM, Ferriani RA, Pfrimer K, Vieira CS. Immediate postpartum initiation of etonogestrel-releasing implant: A randomized controlled trial on breastfeeding impact. Contraception. 2015;92(6):536-42.
54. Cissé AS, Dossou N, Ndiaye M, Guèye AL, Diop EHI, Diaham B, et al. Stable isotope aided evaluation of community nutrition program: Effect of food supplementation schemes on maternal and infant nutritional status. Food and Nutrition Bulletin. 2002;23(3 SUPP):169-73.
55. Galpin L, Thakwalakwa C, Phuka J, Ashorn P, Maleta K, Wong WW, et al. Breast milk intake is not reduced more by the introduction of energy dense complementary food than by typical infant porridge. Journal of Nutrition. 2007;137(7):1828-33.

56. Kumwenda C, Dewey KG, Hemsworth J, Ashorn P, Maleta K, Haskell MJ. Lipid-based nutrient supplements do not decrease breast milk intake of Malawian infants. American Journal of Clinical Nutrition. 2014;99(3):617-23.
57. Lokonon JHF, Hounkpatin WA, Idohou-Dossou N. Participation in the "nutrition at the Centre" project through women's group improved exclusive breastfeeding practices, as measured by the deuterium oxide dose-to-mother technique. International breastfeeding journal. 2020;15(1):58.
58. Mofid LS, Casapía M, Montresor A, Rahme E, Marquis GS, Vercruysse J, et al. Maternal postpartum deworming and infant milk intake: Secondary outcomes from a trial. Maternal and Child Nutrition. 2021;17(4).
59. Moore SE, Prentice AM, Coward WA, Wright A, Frongillo EA, Fulford AJC, et al. Use of stable-isotope techniques to validate infant feeding practices reported by Bangladeshi women receiving breastfeeding counseling. American Journal of Clinical Nutrition. 2007;85(4):1075-82.
60. Owino VO, Kasonka LM, Sinkala MM, Wells JK, Eaton S, Darch T, et al. Fortified complementary foods with or without alpha-amylase treatment increase hemoglobin but do not reduce breast milk intake of 9-mo-old Zambian infants. Am J Clin Nutr. 2007;86(4):1094-103.
61. Owino VO, Bahwere P, Bisimwa G, Mwangi CM, Collins S. Breast-milk intake of 9-10-mo-old rural infants given a ready-to-use complementary food in South Kivu, democratic Republic of Congo. American Journal of Clinical Nutrition. 2011;93(6):1300-4.
62. Wells JC, Jonsdottir OH, Hibberd PL, Fewtrell MS, Thorsdottir I, Eaton S, et al. Randomized controlled trial of 4 compared with 6 mo of exclusive breastfeeding in Iceland: differences in breast-milk intake by stable-isotope probe. Am J Clin Nutr. 2012;96(1):73-9.
63. Salkind NJ. Encyclopedia of Research Design. United kingdom SAGE Publications; 2010.
64. Warner RM. Applied Statistics II: Multivariable and Multivariate Techniques. United states SAGE Publications; 2020.
